# P17. RIG-I-like helicases induce immunogenic cell death of pancreatic cancer cells

**DOI:** 10.1186/2051-1426-2-S2-P8

**Published:** 2014-03-12

**Authors:** M Schnurr, A Steger, H Lohr, H Bourhis, S Endres, P Duewell

**Affiliations:** 1 Division of Clinical Pharmacology, Munich, Germany; 2 LMU Munich, Division of Clinical Pharmacology, Munich, Germany

## Background

We recently identified RIG-I-like helicases (RLH) as therapeutic targets of pancreatic cancer for counteracting immunosuppressive mechanisms and apoptosis induction. Here, we investigated immunogenic consequences of RLH-induced tumour cell death.

## Material and methods

Murine pancreatic cancer cells (Panc02) were treated with RLH ligands to induce apoptosis and were then cocultured with primary dendritic cells (DC). DC maturation marker expression, antigen uptake and antigen cross-presentation were assessed.

## Results

RLH ligands induced production of type I IFN, HMGB1 and Hsp70 and translocation of calreticulin to the outer cell membrane of tumour cells. In cocultures, DC upregulated B7 expression, which was mediated by tumour-derived type I IFN, whereas TLR, RAGE or inflammasome signaling was dispensable. CD8a^+^ DC effectively engulfed apoptotic tumour material and cross-presented tumour-associated antigen to naïve CD8^+^ T cells. In comparison, tumour cell death mediated by oxaliplatin, staurosporine or mechanical disruption failed to induce DC activation, antigen uptake or cross-presentation. Moreover, tumour cells treated with sublethal doses of RLH ligands upregulated MHC-I and Fas expression and were sensitised towards CTL- and Fas-mediated killing.

## Conclusions

RLH ligands induce a highly immunogenic form of tumour cell death linking innate and adaptive immunity.

